# The Effect of CT Scan Parameters on the Measurement of CT Radiomic Features: A Lung Nodule Phantom Study

**DOI:** 10.1155/2019/8790694

**Published:** 2019-02-06

**Authors:** Young Jae Kim, Hyun-Ju Lee, Kwang Gi Kim, Seung Hyun Lee

**Affiliations:** ^1^Department of Biomedical Engineering, Gachon University College of Medicine, Incheon, Republic of Korea; ^2^Department of Plazma Bio Display, Kwangwoon University, Seoul, Republic of Korea; ^3^Department of Radiology, Seoul National University Hospital, Seoul, Republic of Korea

## Abstract

The purpose of this study was to explore the effects of CT slice thickness, reconstruction algorithm, and radiation dose on quantification of CT features to characterize lung nodules using a chest phantom. Spherical lung nodule phantoms of known densities (−630 and + 100 HU) were inserted into an anthropomorphic thorax phantom. CT scan was performed ten times with relocations. CT data were reconstructed using 12 different imaging settings; three different slice thicknesses of 1.25, 2.5, and 5.0 mm, two reconstruction kernels of sharp and standard, and two radiation dose of 30 mAs and 12 mAs. Lesions were segmented using a semiautomated method. Twenty representative CT quantitative features representing CT density and texture were compared using multiple regression analysis. In 100 HU nodule phantoms, 18 and 19 among 20 computer features showed significant difference between different mAs and reconstruction algorithms, respectively (*p* ≤ 0.05). 20, 19, and 19 computer features showed difference between slice thickness of 5.0 vs 1.25, 5.0 vs 2.5, and 2.5 vs 1.25 mm, respectively (*p* ≤ 0.05). In −630 HU nodule phantoms, 18 and 19 showed significant difference between different mAs and reconstruction algorithms, respectively (*p* ≤ 0.05). 18, 11, and 17 computer features showed difference between slice thickness of 5.0 vs 1.25, 5.0 vs 2.5, and 2.5 vs 1.25 mm, respectively (*p* ≤ 0.05). When comparing the absolute value of regression coefficient, the effect of slice thickness in 100 HU nodule and reconstruction algorithm in −630 HU nodule was greater than the effect of remaining scan parameters. The slice thickness, mAs, and reconstruction algorithm had a significant impact on the quantitative image features. In clinical studies involving deep learning or radiomics, it should be noted that differences in values can occur when using computer features obtained from different CT scan parameters in combination. Therefore, when interpreting the statistical analysis results, it is necessary to reflect the difference in the computer features depending on the scan parameters.

## 1. Introduction

Biomedical images may contain information that reflects underlying pathophysiology of many diseases. Nowadays, based on high-throughput computing, extracting many quantitative features from tomographic images is possible. Therefore, many studies have focused on how to convert information on images to quantitative computer features. The conversion of digital medical images into high-dimensional computer data is known as radiomics. Radiomics is also a decision support tool, and it can involve combining radiomic data with other patient characteristics such as survival and disease phenotype [1, 2].

Computer features based on computed tomography (CT) histogram and texture are most frequently used for the differential diagnosis of various cancers including lung cancer [3–6]. Histogram features represent the density of lung nodules and are indicators of distribution of attenuation. On the other hand, digital images are formed from pixels that are too small to be recognized by the human eye. However, the human visual system can detect the patterns such as roughness and smoothness. Such spatial variation of pixel intensities can be represented as the texture. Computerized analysis of a pattern of brightness and darkness is called texture analysis, and texture features show surface information by examining the relationship between voxels on images [7, 8].

Recently, the number of researches on radiomics or deep learning is increasing [9–14]. Scan parameters can affect the image quality or noise, and some of the computer features extracted from these images are at risk of varying values. Nevertheless, imaging data from clinical studies are usually obtained using different scan parameters. For accurate analysis, images acquired with different parameters should be normalized to the same conditions. For normalization, it is necessary to quantitatively analyze the magnitude of the effects of the change of the scan parameter on the feature values. Several studies have been reported on the quantification of characteristic values according to scan parameters. Zhao et al. [15] reported differences in radiomic features due to changes in slice thickness and reconstruction algorithms. The repeated CT data were collected from 32 lung cancer patients, and experiments were performed on the reproducibility of radiomic features in 6 different combinations of slice thickness and reconstruction algorithms. Also, differences in radiomic features due to changes in reconstruction algorithms at the same slice thickness were analyzed. As a result, the radiomic features were reproducible when the CT was obtained repeatedly under the same conditions. However, Zhao et al. reported that the radiomic features differed in the change of the reconstruction algorithm. Mackin et al. [16] reported the effect of tube current on radiomic features using the phantom in CT. The differences between 48 features extracted from 25 mAs to 300 mAs conditions were analyzed in the CCR phantom, which was made with 10 textures. As a result, the changes in the tube current influenced more on the features extracted from homogeneous materials (acrylic, sycamore wood) than materials with more tissue-like textures (cork, rubber particles). Mackin et al. reported that tube currents do not have a significant effect on the radiomic features extracted from the tissue texture such as tumors. Kim et al. [17] also reported the tube current effect on radiomic features in CT. A total of 15 features were extracted from 42 CT data based on two reconstruction algorithms, filtered back projection, and iterative reconstruction algorithm, respectively. The difference between the features extracted from the two reconstruction algorithms was analyzed. As a result, Kim et al. reported that 9 of 15 features showed significant differences. Several studies have been reported on the differences in radiomic features for changes in scan parameters. However, most of the studies were analyzed using one scan parameter. We need to analyze the relationship between more various scan parameters and radiomic features.

Therefore, the purpose of this study was to analyze the effect of various scan parameters on the quantitative CT features of lung nodule phantoms. We evaluated the effect of different CT slice thicknesses, mAs, and reconstruction algorithms on 3-dimensional computer features including CT histogram, gray-level co-occurrence matrix (GLCM), and gray-level run length matrix (GLRLM).

## 2. Materials and Methods

### 2.1. Lung Nodules

**Figure 1:**
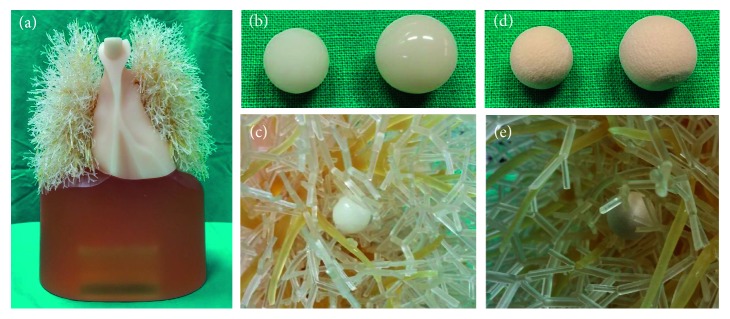
Anthropomorphic thorax phantom and nodule phantoms: (a) chest phantom, (b) nodule phantoms of 100 HU, (c) nodule phantoms of −630 HU, (d) an example of a 100 HU nodule phantom attached to the pulmonary vasculature, and (e) an example of a −630 HU nodule phantom attached to the pulmonary vasculature.

In this study, we used an anthropomorphic thorax phantom (KYOTO KAGAKU co., Kyoto, Japan) and nodule phantoms of two different attenuation values (100 Hounsfield Unit (HU) and −630 HU) (Figure 1). The pulmonary nodule can be divided into a solid component and a ground glass component depending on the component. It is generally known that the solid component has an attenuation value of 100 HU, and the ground glass component has an attenuation value of  630 HU. Therefore, we used two types of nodal phantoms with attenuation values of 100 HU and −630 HU on CT. The 100 HU nodules were made with polyurethane and hydroxyapatite, and the −630 HU nodules were made with urethane foam. The size of nodule phantoms was 10 mm and 12 mm in each attenuation value [18]. So, total four kinds of nodule phantoms were used. We inserted nodule phantoms into the two lungs (2 lesions per lung).

### 2.2. Image Acquisition

**Figure 2:**
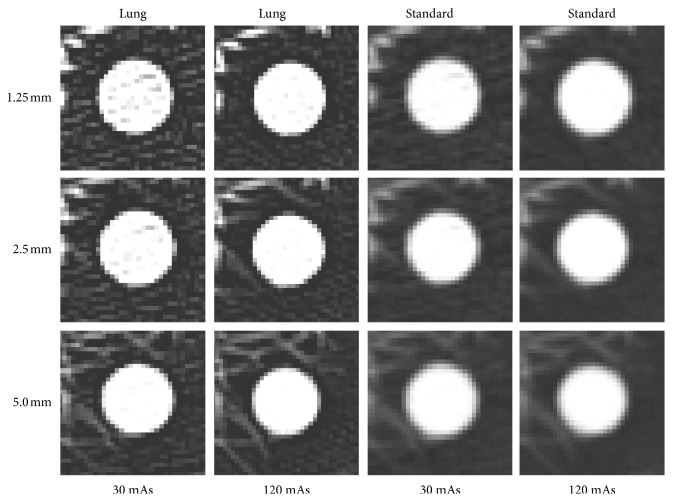
CT images of a 12 mm sized 100 HU nodule phantom in 12 different scan parameters.

**Figure 3:**
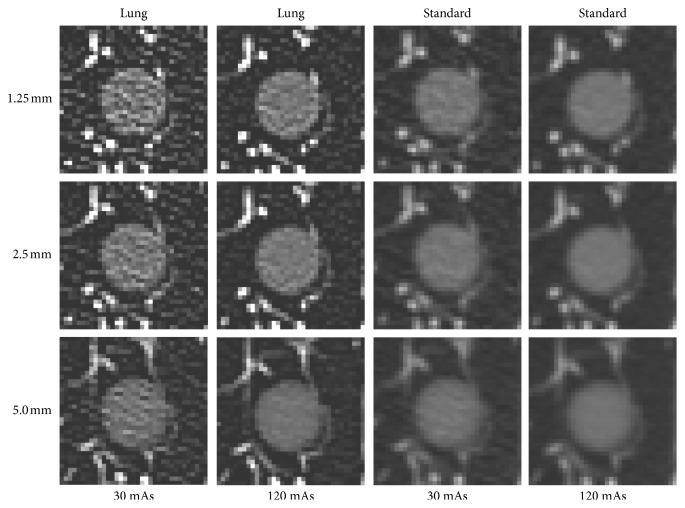
CT images of a 12 mm sized −630 HU nodule phantom in 12 different scan parameters.

CT scan was performed by using a 64 channel multi-detector row CT scanner (GE Discovery CT 750 HD; GE Healthcare, USA). The CT scan parameters were 120 kVp, 64 × 0.625 collimator configuration, and pitch of 0.984 : 1. The raw data were then reconstructed using 12 different combinations of scan parameters; slice thicknesses (1.25, 2.5, 5.0), mAs (30, 120), and reconstruction algorithms (lung, standard). In each combination of scan parameter, CT scan was repeated 10 times with relocation of nodule phantoms (Figures 2 and 3).

**Figure 4:**
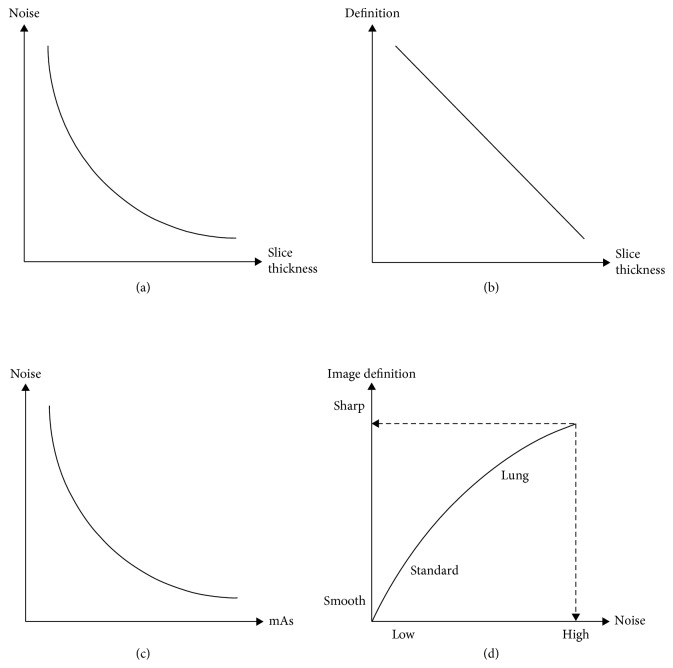
Relationship between scan parameters and image quality: (a) relationship between slice thickness variation and noise, (b) relationship between slice thickness variation and definition, (c) relationship between mAs variation and noise, and (d) relationship between reconstruction algorithms and noise definition.

In general, it is known that definition and noise decreases as slice thickness increases, noise decreases as mAs increases, and the standard algorithm has less definition and noise than the lung algorithm.  Figure 4 shows a graph of the relationship between each scan parameter and noise, sharpness.

### 2.3. Nodules Segmentation

In this study, we used an in-house software for the computerized analysis of CT images. This software was developed by using Microsoft Visual Studio (Ver. 2010, Microsoft, Redmond, WA, USA), ITK (Insight Segmentation and Registration Toolkit, Kitware Inc., NY, USA), and VTK (Visualization Toolkit, Kitware Inc., NY, USA). For the first step of the volume measurement of the nodules, the entire tumor mass was separated from surrounding anatomic structures by using a semiautomated segmentation algorithm developed in the Laboratory for Computational Image Analysis in the Department of Biomedical Engineering of Gachon University College of Medicine. This algorithm combined the image analysis techniques of seed region-growing algorithm [19]. Computer-generated tumor boundaries were then visually inspected by a radiologist (HJL, with 19 years of experience performing chest image interpretations) for correctness and consistency. If any segmentation results were considered suboptimal, tumor contours were edited by the same radiologist (HJL).

### 2.4. Features Selection and Extraction

In this study, we used the features mainly used in lung nodule analysis. Among the various features, 20 radiomic features were selected based on several related papers that performed lung nodule analysis [20–34].

In this study, we quantized the pixel values of 4,096 gray colors into 256 gray colors, and texture features are extracted based on the discretized pixel values [35]. A total of 20 computer features including 7 histogram features and 13 texture features were extracted from each nodule phantom (Table 1). Histogram features were mean of CT attenuation, standard deviation (stddev), variance, skewness, kurtosis, energy, and entropy. GCLM texture features were contrast, dissimilarity, homogeneity, angular second moment (ASM), energy, probability max, entropy, and correlation. GLRLM texture features were long runs emphasis (LRE), gray-level nonuniformity (GLN), run length nonuniformity (RLN), low-gray-level run emphasis (LGRE), and high-gray-level run emphasis (HGRE) [7, 8, 36–39].

GLCM is a matrix that represents the frequency of occurrence in the relationship of gray level between neighboring voxels with a specific direction. GLRLM is a matrix characterized by the frequency of occurrence in the consecutive voxels with the same attenuation value along a specific direction [8].

**Figure 5:**
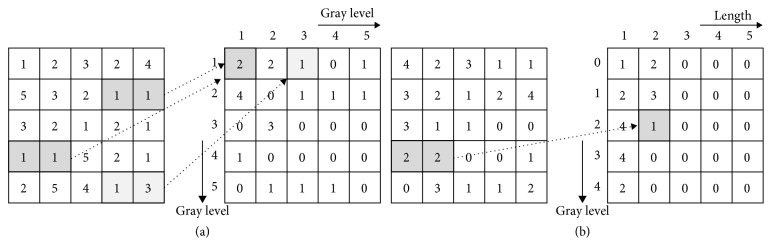
The calculation process of (a) GLCM and (b) GLRLM (distance: 1, direction: 0°).

The GLCM is represented by the maximum size of the gray level, both in rows and columns. The relationship between gray values of all the pixels and the gray values of neighboring pixels according to a given direction and distance is expressed in the number of occurrences in the matrix. In Figure 5(a), the number of occurrences is 2 when the gray value of the target pixel in the left image is 1 and the gray value of the neighboring pixel under the given condition is 1. Therefore, (1, 1) in the right GLCM becomes the total number of occurrences, 2.

In the GLRLM, the rows are represented by gray values and the columns are expressed by the same number of adjacent pixels. The number of occurrences for the case where the gray value of each of the pixels is the same as the gray value of the neighboring pixels according to a given direction, and distance is represented by a matrix. In Figure 5(b), the number of occurrences is 1 when the gray value of the target pixel in the left image is 2 and the length of the same gray value of the neighboring pixel under the given condition is 2. Therefore, (2, 2) in the right GLRLM becomes the total number of occurrences, 1.

**Figure 6:**
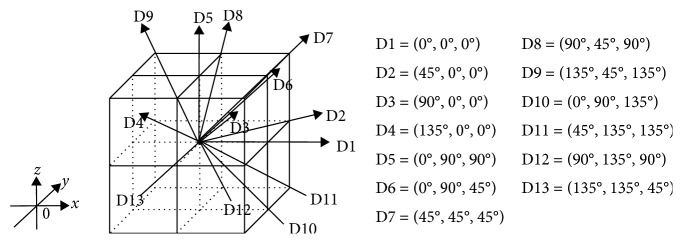
13 Directions for matrix calculation on a three-dimensional space. On a three-dimensional space, GLCM and GLRLM can generally conduct a matrix calculation in 13 directions.

On a three-dimensional space, GLCM and GLRLM can generally conduct a matrix calculation in 13 directions (Figure 6). The calculation of GLCM and GLRLM values was conducted in each of 13 directions, and we used the mean value of each calculation for statistical analysis [40, 41]. The distance between voxels was set as 1. The computer features were calculated from the equations in Table 1 in the GLCM and the GLRLM.

### 2.5. Statistical Analysis

Multiple regression analysis was performed to evaluate the effect of different scan parameters on the computer features of nodule phantoms (SPSS version 18.0, SPSS Inc., USA) [42, 43]. The dependent variables were 20 computer features, and independent variables were slice thickness (three variables), mAs (two variables), and reconstruction algorithm (two variables). For the statistical analysis, categorical variables were converted into dummy variables. We assumed that independent variables have a linear relationship with dependent variables. We performed an absolute effect size analysis to evaluate the difference in the change of the computer features according to the scan parameter. We calculated Cohen's d effect size by dividing the mean difference by their pooled standard deviation.

## 3. Results

The results of multiple regression analysis in 100 HU nodule phantoms are presented in Tables 2 and 3. In 95 sets of parameter comparison among 100 sets of comparison, computer features showed significant difference. Between different slice thicknesses, 19 computer features showed significant difference (*p* ≤ 0.05). The only feature, dissimilarity, showed no difference between slice thicknesses of 5.0 mm and 2.5 mm (*p*=0.437) and also showed no difference between slice thicknesses of 2.5 mm and 1.25 mm (*p*=0.572). Between 30 mAs and 120 mAs, 18 computer features showed significant difference (*p* ≤ 0.05). Two features including kurtosis (*p*=0.217) and LGRE (*p*=0.19) showed no difference. Between different reconstruction algorithms, 19 computer features showed significant difference (*p* ≤ 0.05), and correlation showed no difference (*p*=0.11). In the absolute effect size analysis, the numbers of large effects, medium effects, and small effects were 19, 1, and 0 between 5.0 mm and 1.25 mm slice thickness, and 18, 1, and 1 between 5.0 mm and 2.5 mm slice thickness, and 15, 4, and 1 between 2.5 mm and 1.25 mm slice thickness, respectively. The numbers of large effects, medium effects, and small effects were 0, 13, and 7 between 30 mAs and 120 mAs, and 2, 16, and 2 between lung and standard reconstruction algorithms, respectively.

The results of multiple regression analysis in −630 HU nodule phantoms are presented in Tables 4 and 5. In 83 sets of parameter comparison among 100 sets of comparison, computer features showed significant difference. Eighteen computer features between slice thicknesses of 5.0 mm and 1.25 mm, 11 features between of 5.0 mm and 2.5 mm, and 17 features between 2.5 mm and 1.25 mm showed significant difference (*p* ≤ 0.05). Between 30 mAs and 120 mAs, 18 computer features showed significant difference (*p* ≤ 0.05). Two features including mean attenuation (*p*=0.163) and LGRE (*p*=0.054) showed no difference. Between different reconstruction algorithms, 19 computer features showed significant difference (*p* ≤ 0.05), and LGRE showed no difference (*p*=0.238). In the absolute effect size analysis, the number of large effects, medium effects, and small effects was 7, 11, and 2 between 5.0 mm and 1.25 mm slice thickness, and 6, 6, and 8 between 5.0 mm and 2.5 mm slice thickness, and 0, 18, and 2 between 2.5 mm and 1.25 mm slice thickness, respectively. The number of large effects, medium effects, and small effects were 6, 12, and 2 between 30 mAs and 120 mAs, and 19, 0, and 1 between lung and standard reconstruction algorithms, respectively.

In the regression analysis, the absolute value of regression coefficient (|RC|) can represent the scale of difference. |RC|s in all features in comparison between 5.0 mm and 1.25 mm were larger than |RC|s between 5.0 mm and 2.5 mm (Tables 2 and 4).

In 100 HU nodule phantoms, the maximum (Max), median (Med), and minimum (Min) values of |RC| were 0.541, 0.388, and 0.011 between 5.0 mm and 2.5 mm slice thickness, and 0.672, 0.574, and 0.135 between 5.0 mm and 1.25 mm slice thickness, respectively. Max, Med, and Min values of |RC| were 0.186, 0.079, and 0.011 between 30 mAs and 120 mAs and 0.271, 0.127, and 0.015 between lung and standard reconstruction algorithms, respectively (Table 2).

In −630 HU nodule phantoms, Max, Med, and Min values of |RC| were 0.247, 0.039, and 0.004 between 5.0 mm and 2.5 mm slice thickness, and 0.277, 0.122, and 0.018 between 5.0 mm and 1.5 mm slice thickness, respectively. Max, Med, and Min values of |RC| were 0.196, 0130, and 0.011 between 30 mAs and 120 mAs and 0.436, 0.224, and 0.011 between lung and standard reconstruction algorithms, respectively (Table 4).

The values of computer features in different slice thicknesses, mAs, and reconstruction algorithms are presented in Supplementary Materials 1 and 2. The computer features in 12 different sets of scan parameters are presented in Supplementary Materials 3 and 4.

## 4. Discussion

Our study showed that (a) in both of 100 HU and −630 HU nodule phantoms, differences in the scan parameters had a significant effect on almost all computer features with few exceptions, (b) in the 100 HU nodule phantoms, considering the Max and Med values of |RC|s between different slice thicknesses were larger than the Max and Med values of |RC|s between different mAs or algorithms, we speculate slice thickness had a greater effect than mAs or algorithm, and (c) in the −630 HU nodule phantoms, considering the Max and Med values of |RC|s between different algorithms were larger than the Max and Med values of |RC|s between different slice thicknesses or mAs, we speculate algorithm had a greater effect than slice thickness or mAs.

In this study, differences in the scan parameters had a significant effect on almost all computer features. These results indicate that noise or artifacts affected the attenuation and texture in the nodule, which indicates that the scan parameters are related to noise and artifact. Also, our results are consistent with several previous studies [38, 44, 45].

In the regression analysis, the |RC| can represent the scale of difference in feature values in different scan parameters. We found in this study that |RC|s in all features in comparison between 5.0 mm and 1.25 mm were larger than |RC|s between 5.0 mm and 2.5 mm. As the slice thickness becomes thinner, the noise increases. As the slice thickness becomes thicker, the noise decreases and greater effect of the partial volume effect [46]. Thus, a large difference in slice thickness will increase the impact of noise and partial volume effect. So it is considered that RC is shown larger in the comparison between 5.0 mm and 1.25 mm.

In the 100 HU nodule phantoms, we found slice thickness had a greater effect than mAs or algorithm. The CT image of 100 HU nodule phantoms has smaller noise than CT of −630 HU nodule phantom due to higher average attenuation and a smaller variation in the distribution of attenuation. Therefore, the 100 HU nodule phantom is more affected by the partial volume effect than the −630 HU nodule phantom. As the result, the slice thickness that is closely related to partial volume effect was the most influential parameter in the 100 HU nodule phantom.

We also found in this study that, in the 100 HU nodule phantoms, GLCM-dissimilarity showed no difference between slice thickness of 5.0 and 2.5 mm and between 2.5 mm and 1.25 mm. The weight of the dissimilarity increases linearly unlike other texture features which increases exponentially [47]. In the features in which the weights increase exponentially, a large difference may occur even if the difference of the image values is small. Since the weight of the dissimilarity increases linearly, the difference is relatively small when compared with other feature values. Therefore, the dissimilarity showed not statistically significant when difference of slice thickness was small such as 5.0 mm and 2.5 mm, and 2.5 mm and 1.25 mm, respectively.

In the −630 HU nodule phantoms, the reconstruction algorithm had a greater effect than slice thickness or mAs. The amount of noise in the −630 HU nodule phantom is greater than the amount in the 100 HU nodule phantom. The lung reconstruction algorithm usually makes higher noise level that the standard algorithm. The change of reconstruction algorithm from standard to lung algorithm made bigger increase of noise in the nodule with inherently higher level of noise, that is, −630 HU nodule.

In this study, in the −630 HU nodule phantoms, the effect of slice thickness was smaller than the effect of the reconstruction algorithm. We can notice that smaller difference of slice thickness might make smaller difference of noise, thus the statistical difference was not significant. On the other hand, due to higher average attenuation and a smaller variation in the distribution of attenuation, the CT image of 100 HU nodule phantoms was more affected by the difference of slice thickness that cause difference of partial volume effect.

CT images of the lung reconstruction algorithm contain higher noise level that the image of standard algorithm. Therefore, the change of the reconstruction algorithm can affect the features associated with CT histogram or CT texture. In this study, we found that change of the algorithms had significant effect on 19 computer features in the 100 and −630 HU nodule phantoms. Only a feature, that is, correlation, in 100 HU phantoms and LGRE in −630 HU nodule phantom showed no difference between lung and standard algorithms.

In this study, we found that most computer features showed significant difference between 30 mAs and 120 mAs. We speculate that this significant difference was originated by the change of noise level. We also found that LGRE showed no difference between 30 mAs and 120 mAs in both of 100 HU and −630 HU nodule phantoms. LGRE, that is, low gray level run emphasis, can be defined by distribution of run length in the low gray values. The value of LGRE is high when there is the large number of pixels with low gray level [37]. Considering the result that LGRE showed no difference between 30 mAs and 120 mAs, the number of pixels with low gray level has not significantly changed by the change of mAs.

This study demonstrated that the change of CT scan parameters can affect the quantitative CT features. In clinical studies involving deep learning or radiomics, it should be noted that differences in values can occur when using computer features obtained from different CT scan parameters in combination. Therefore, when interpreting the statistical analysis results, it is necessary to reflect the difference in the computer features depending on the scan parameters. In further studies, we need to develop methods for the standardization of computer features obtained from different scan parameters.

## Figures and Tables

**Table 1:** Definition of the 20 computer features.

Features		Definition	Description
Histogram	Mean	(1/*N*)∑_*i*=0_^*N*−1^∑_*j*=0_^*N*−1^ *P*_*i*,*j*_	The mean value of the histogram distribution
	Stddev	$\sqrt{\left(1/N\right)\sum_{i=0}^{N-1}\sum_{j=0}^{N-1}{\left(P_{i,j}-\bar{P}\right)}^{2}}$	The square root of the variance
	Variance	$\left(1/N\right)\sum_{i=0}^{N-1}\sum_{j=0}^{N-1}{\left(P_{i,j}-\bar{P}\right)}^{2}$	The amount of variation of the histogram distribution
	Skewness	$\left(\left(\left(1/N\right)\sum_{i=0}^{N-1}\sum_{j=0}^{N-1}{\left(P_{i,j}-\bar{P}\right)}^{3}\right)/\left({\left(\left(1/N\right)\sum_{i=0}^{N-1}\sum_{j=0}^{N-1}{\left(P_{i,j}-\bar{P}\right)}^{2}\right)}^{3/2}\right)\right)$	The asymmetry of the histogram distribution
	Kurtosis	$\left(\left(\left(1/N\right)\sum_{i=0}^{N-1}\sum_{j=0}^{N-1}{\left(P_{i,j}-\bar{P}\right)}^{4}\right)/\left({\left(\left(1/N\right)\sum_{i=0}^{N-1}\sum_{j=0}^{N-1}{\left(P_{i,j}-\bar{P}\right)}^{2}\right)}^{2}\right)\right)-3$	The flatness of the histogram distribution
	Energy	∑_*i*=0_^*N*−1^∑_*j*=0_^*N*−1^[*P*_*i*,*j*_]^2^	The uniformity of the histogram distribution
	Entropy	∑_*i*=0_^*N*−1^∑_*j*=0_^*N*−1^ *P*_*i*,*j*_ log_2_[*P*_*i*,*j*_]^2^	The randomness of the histogram distribution

LCM	Contrast	∑_*i*=0_^*N*−1^∑_*j*=0_^*N*−1^ *P*_*i*,*j*_(*i* − *j*)^2^	The local variation of voxel pairs
	Dissimilarity	∑_*i*=0_^*N*−1^∑_*j*=0_^*N*−1^ *P*_*i*,*j*_|*i* − *j*|	The variation of voxel pairs
	Homogeneity	∑_*i*=0_^*N*−1^∑_*j*=0_^*N*−1^((*P*_*i*,*j*_)/(1+(*i* − *j*)^2^))	The homogeneity of voxel pairs
	Angular second moment (ASM)	∑_*i*=0_^*N*−1^∑_*j*=0_^*N*−1^ *P*_*i*,*j*_^2^	The uniformity of voxel pairs
	Energy	$\sqrt{\sum_{i=0}^{N-1}\sum_{j=0}^{N-1}P_{i,j}^{2}}$	Square root of the ASM
	Probability max	max(*P*_*i*,*j*_)	High max value of voxel pairs
	Entropy	−∑_*i*=0_^*N*−1^∑_*j*=0_^*N*−1^ *P*_*i*,*j*_(log_2_ *P*_*i*,*j*_)	The randomness of voxel pairs
	Correlation	∑_*i*=0_^*N*−1^∑_*j*=0_^*N*−1^ *P*_*i*,*j*_[(((*i* − *μ*_*i*_)(*j* − *μ*_*j*_))/(*σ*_*i*_ *σ*_*j*_))]	The linear dependency of gray levels

GLRLM	Long runs emphasis (LRE)	∑_*i*=0_^*G*−1^∑_*j*=0_^*R*−1^ *j* ^2^ *P*_*i*,*j*_	The distribution of the long run length
	Gray-level nonuniformity (GLN)	∑_*i*=0_^*G*−1^(∑_*j*=0_^*R*−1^ *P*_*i*,*j*_)^2^	The nonuniformity of the gray level
	Run length nonuniformity (RLN)	∑_*i*=0_^*R*−1^(∑_*j*=0_^*G*−1^ *P*_*i*,*j*_)^2^	The nonuniformity of the run length
	Low-gray-level run emphasis (LGRE)	∑_*i*=0_^*G*−1^∑_*j*=0_^*R*−1^ *P*_*i*,*j*_/*i* ^2^	The distribution of the low gray level groups
	High-gray-level run emphasis (HGRE)	∑_*i*=0_^*G*−1^∑_*j*=0_^*R*−1^ *i* ^2^ *P*_*i*,*j*_	The distribution of the high gray level groups

**Table 2:** Regression coefficients in the comparison between different slice thickness, mAs, and reconstruction algorithm in 100 HU nodule phantoms.

Image features			Constant	Slice thickness			mAs		Reconstruction algorithm	
				5.00 (ref.)	2.50	1.25	30 (ref.)	120	Lung (ref.)	Standard
Histogram		Mean	0.323	0	0.418^†^	0.613^†^	0	0.020^†^	0	−0.271^†^
		Stddev	0.442	0	−0.085^†^	−0.237^†^	0	−0.055^†^	0	−0.075^†^
		Variance	0.260	0	−0.058^†^	−0.152^†^	0	−0.036^†^	0	−0.047^†^
		Skewness	0.008	0	0.179^†^	0.463^†^	0	0.051^†^	0	0.108^†^
		Kurtosis	0.772	0	−0.135^†^	−0.177^†^	0	−0.032	0	−0.234^†^
		Energy	0.112	0	0.388^†^	0.642^†^	0	0.103^†^	0	−0.209^†^
		Entropy	0.814	0	−0.430^†^	−0.664^†^	0	−0.079^†^	0	0.188^†^

GLCM		Contrast	0.078	0	0.171^†^	0.383^†^	0	−0.022^†^	0	−0.026^†^
		Dissimilarity	0.231	0	0.011	0.135^†^	0	−0.058^†^	0	0.036^†^
		Homogeneity	0.147	0	0.472^†^	0.600^†^	0	0.131^†^	0	−0.127^†^
		ASM	0.025	0	0.387^†^	0.552^†^	0	0.186^†^	0	−0.159^†^
		Energy	0.130	0	0.476^†^	0.614^†^	0	0.142^†^	0	−0.145^†^
		Probability max	0.150	0	0.466^†^	0.583^†^	0	0.141^†^	0	−0.137^†^
		Entropy	0.856	0	−0.454^†^	−0.646^†^	0	−0.116^†^	0	0.153^†^
		Correlation	0.927	0	−0.129^†^	−0.299^†^	0	0.026^†^	0	0.015

GLRLM		LRE	0.036	0	0.452^†^	0.574^†^	0	0.178^†^	0	−0.097^†^
		GLN	0.105	0	0.418^†^	0.621^†^	0	0.117^†^	0	−0.181^†^
		RLN	0.745	0	−0.541^†^	−0.636^†^	0	−0.105^†^	0	0.116^†^
		LGRE	0.212	0	−0.130^†^	−0.180^†^	0	−0.011	0	0.037^†^
		HGRE	0.288	0	0.509^†^	0.672^†^	0	0.039^†^	0	−0.161^†^

^†^ *p* ≤ 0.05.

**Table 3:** Absolute effect size in 100 HU phantom nodules.

Image features			Slice thickness						mAs		Reconstruction algorithm	
			5.00 vs 1.25		5.00 vs 2.50		2.50 vs 1.25		30 vs 120		Lung vs standard	
			Effect	*d*	Effect	*d*	Effect	*d*	Effect	*d*	Effect	*d*
Histogram		Mean	<	4.62	<	2.81	<	1.18	<	0.07	>	1.02
		Stddev	>	3.06	>	1.05	>	1.40	>	0.42	>	0.59
		Variance	>	3.35	>	0.72	>	1.06	>	0.38	>	0.50
		Skewness	<	1.98	<	1.77	<	1.20	<	0.18	<	0.40
		Kurtosis	>	0.64	>	1.58	>	0.15	0	0.13	>	1.09
		Energy	<	5.63	<	2.70	<	1.44	<	0.35	>	0.74
		Entropy	>	7.06	>	3.25	>	1.67	>	0.26	<	0.65

GLCM		Contrast	<	5.73	<	2.27	<	2.26	>	0.13	>	0.15
		Dissimilarity	<	2.04	0	0.11	0	1.05	>	0.52	<	0.31
		Homogeneity	<	5.81	<	3.36	<	0.87	<	0.46	>	0.45
		ASM	<	4.20	<	2.32	<	0.79	<	0.68	>	0.57
		Energy	<	5.53	<	3.28	<	0.88	<	0.49	>	0.50
		Probability max	<	5.25	<	3.28	<	0.77	<	0.51	>	0.49
		Entropy	>	6.56	>	3.38	>	1.34	>	0.39	<	0.53
		Correlation	>	5.61	>	1.69	>	1.96	<	0.18	0	0.10

GLRLM		LRE	<	4.40	<	3.05	<	0.65	<	0.64	>	0.34
		GLN	<	5.36	<	2.96	<	1.17	<	0.40	>	0.64
		RLN	>	5.35	>	3.85	>	0.88	>	0.35	<	0.39
		LGRE	>	5.18	>	1.59	>	0.64	0	0.11	<	0.37
		HGRE	<	6.30	<	3.93	<	1.66	<	0.13	>	0.54

<  indicates *p* ≤ 0.05 and A is statistically smaller than B (A vs B); > indicates *p* ≤ 0.05 and A is statistically larger than B (A vs B); 0 indicates A and B are not statistically significant (A vs B). Cohen's *d*: small ≥ 0.20; medium ≥ 0.50; large ≥ 0.80.

**Table 4:** Regression coefficients in the comparison between different slice thickness, mAs, and reconstruction algorithm in −630 HU nodule phantoms.

Image features			Constant	Slice thickness			mAs		Reconstruction algorithm	
				5.00 (ref.)	2.50	1.25	30 (ref.)	120	Lung (ref.)	Standard
Histogram		Mean	0.617	0	0.247^†^	0.277^†^	0	−0.011	0	−0.154^†^
		Stddev	0.369	0	0.036	0.158^†^	0	−0.149^†^	0	−0.279^†^
		Variance	0.230	0	0.034	0.154^†^	0	−0.125^†^	0	−0.200^†^
		Skewness	−0.023	0	0.209^†^	0.256^†^	0	0.143^†^	0	0.224^†^
		Kurtosis	0.985	0	−0.146^†^	−0.098^†^	0	−0.149^†^	0	−0.436^†^
		Energy	0.061	0	0.063^†^	−0.021	0	0.189^†^	0	0.361^†^
		Entropy	0.664	0	0.014	0.122^†^	0	−0.196^†^	0	−0.394^†^

GLCM		Contrast	0.237	0	0.111^†^	0.238^†^	0	−0.108^†^	0	−0.206^†^
		Dissimilarity	0.349	0	0.121^†^	0.251^†^	0	−0.141^†^	0	−0.292^†^
		Homogeneity	0.193	0	−0.039^†^	−0.154^†^	0	0.191^†^	0	0.422^†^
		ASM	0.027	0	−0.004	−0.060^†^	0	0.109^†^	0	0.166^†^
		Energy	0.080	0	−0.015	−0.086^†^	0	0.139^†^	0	0.233^†^
		Probability max	0.052	0	−0.008	−0.036^†^	0	0.084^†^	0	0.122^†^
		Entropy	0.788	0	0.042	0.130^†^	0	−0.153^†^	0	−0.284^†^
		Correlation	0.752	0	−0.100^†^	−0.228^†^	0	0.109^†^	0	0.204^†^

GLRLM		LRE	0.068	0	0.014	−0.054^†^	0	0.130^†^	0	0.258^†^
		GLN	0.033	0	0.033^†^	−0.018	0	0.125^†^	0	0.223^†^
		RLN	0.891	0	−0.004	0.078^†^	0	−0.163^†^	0	−0.318^†^
		LGRE	0.167	0	−0.106^†^	−0.086^†^	0	−0.018	0	0.011
		HGRE	0.639	0	0.227^†^	0.269^†^	0	−0.030^†^	0	−0.144^†^

^†^ *p* ≤ 0.05.

**Table 5:** Effect of scan parameters on computer features in −630 HU phantom nodules.

Image features		Slice thickness						mAs		Reconstruction algorithm	
		5.00 vs 1.25		5.00 vs 2.50		2.50 vs 1.25		30 vs 120		Lung vs standard	
		Effect	*d*	Effect	*d*	Effect	*d*	Effect	*d*	Effect	*d*
Histogram	Mean	<	2.85	<	2.71	<	0.28	0	0.07	>	1.11
	Stddev	<	0.71	0	0.25	<	0.50	>	0.72	>	1.65
	Variance	<	0.74	0	0.35	<	0.55	>	0.68	>	1.20
	Skewness	<	1.15	<	1.25	0	0.19	<	0.61	<	1.04
	Kurtosis	>	0.40	>	0.63	<	0.16	>	0.58	>	2.77
	Energy	0	0.10	<	0.29	>	0.31	<	0.89	<	2.46
	Entropy	<	0.52	0	0.07	<	0.38	>	0.85	>	2.56

GLCM	Contrast	<	1.32	<	0.97	<	0.65	>	0.58	>	1.27
	Dissimilarity	<	1.21	<	0.74	<	0.56	>	0.65	>	1.67
	Homogeneity	>	0.69	>	0.16	>	0.40	<	0.79	<	2.81
	ASM	>	0.48	0	0.03	>	0.42	<	0.87	<	1.53
	Energy	>	0.55	0	0.08	>	0.41	<	0.87	<	1.80
	Probability max	>	0.34	0	0.06	0	0.26	<	0.76	<	1.21
	Entropy	<	0.63	0	0.20	<	0.41	>	0.75	>	1.72
	Correlation	>	1.20	>	0.80	>	0.65	<	0.58	<	1.21

GLRLM	LRE	>	0.40	0	0.08	>	0.34	<	0.79	<	2.12
	GLN	0	0.13	<	0.23	>	0.31	<	0.91	<	2.23
	RLN	<	0.46	0	0.02	<	0.34	>	0.83	>	2.27
	LGRE	>	0.98	>	1.49	0	0.35	0	0.21	0	0.13
	HGRE	<	2.89	<	2.64	<	0.44	>	0.20	>	1.09

<  indicates *p* ≤ 0.05 and A is statistically smaller than B (A vs B); >  indicates *p* ≤ 0.05 and A is statistically larger than B (A vs B); 0 indicates A and B are not statistically significant (A vs B). Cohen's *d*: small ≥ 0.20; medium ≥ 0.50; large ≥ 0.80.

## Data Availability

The data used to support the findings of this study are available from the corresponding author upon request.

## Supplementary Materials

Supplementary Materials Supplementary Material 1: the computer features according to the scanning parameters in the 100 HU nodule phantoms. Supplementary Material 2: the computer features according to the scanning parameters in the −630 HU nodule phantoms. Supplementary Material 3: the computer features in 12 different scan parameters in 100 HU nodule phantoms. Supplementary Material 4: the computer features in 12 different scan parameters in −630 HU nodule phantoms.
