# Supplementary material for: The Effect of CT Scan Parameters on the Measurement of CT Radiomic Features: A Lung Nodule Phantom Study
Source: Comput Math Methods Med. 2019 Feb 6;2019:8790694. doi: 10.1155/2019/8790694 (PMC6381551; doi:10.1155/2019/8790694)
Supplement: Supplementary Materials — Supplementary Material 1: the computer features according to the scanning parameters in the 100 HU nodule phantoms. Supplementary Material 2: the computer features according to the scanning parameters in the −630 HU nodule phantoms. Supplementary Material 3: the computer features in 12 different scan parameters in 100 HU nodule phantoms. Supplementary Material 4: the computer features in 12 different scan parameters in −630 HU nodule phantoms. [file 8790694.f1.docx]

Supplementary material 1. The Computer Features according to the Scanning Parameters in the 100HU Nodule Phantoms.

|  | | Slice Thickness | | |  | mAs | |  | Reconstruction Algorithm | |
| --- | --- | --- | --- | --- | --- | --- | --- | --- | --- | --- |
|  |  | 5.0 | 2.5 | 1.25 |  | 30 | 120 |  | Lung | Standard |
| Histogram | Mean | -121.88±38.99 | 26.58±63.71 | 96.08±54.19 |  | -3.27±107.97 | 3.79±103.14 |  | 48.37±103.49 | -47.85±83.32 |
|  | Stdev | 191.1±8.75 | 164.7±34.58 | 117.23±33 |  | 166.2±39.56 | 149.15±41.72 |  | 169.39±35.23 | 145.96±43.97 |
|  | Variance | 36594.57±3324.54 | 28306.66±15866.87 | 14817.66±8559.65 |  | 29173.36±11779.62 | 23972.56±15269.27 |  | 29924.4±14522.71 | 23221.53±12328.24 |
|  | Skewness | 3.54±1.18 | 5.78±1.34 | 9.33±3.96 |  | 5.9±3.22 | 6.53±3.65 |  | 5.54±1.53 | 6.89±4.55 |
|  | Kurtosis | -0.94±0.31 | -1.42±0.3 | -1.57±1.36 |  | -1.25±0.83 | -1.37±0.89 |  | -0.9±0.57 | -1.72±0.91 |
|  | Energy | 0.06±0.04 | 0.37±0.16 | 0.57±0.12 |  | 0.29±0.2 | 0.37±0.27 |  | 0.41±0.25 | 0.25±0.19 |
|  | Entropy | 5.83±0.47 | 3.39±0.96 | 2.06±0.59 |  | 3.99±1.57 | 3.54±1.83 |  | 3.23±1.71 | 4.3±1.56 |
| GLCM | Contrast | 125.82±22.28 | 232.07±62.26 | 364.38±54.44 |  | 247.72±103.25 | 233.8±115.4 |  | 248.92±113 | 232.6±105.7 |
|  | Dissimilarity | 1.88±0.09 | 1.92±0.42 | 2.29±0.27 |  | 2.12±0.25 | 1.94±0.4 |  | 1.97±0.41 | 2.08±0.26 |
|  | Homogeneity | 0.01452±0.00423 | 0.03586±0.00793 | 0.04162±0.00507 |  | 0.02772±0.01071 | 0.03362±0.01458 |  | 0.03355±0.01331 | 0.02779±0.01228 |
|  | ASM | 0.00011±0.0001 | 0.00111±0.0006 | 0.00154±0.00047 |  | 0.00068±0.00047 | 0.00116±0.00088 |  | 0.00113±0.00081 | 0.00072±0.00061 |
|  | Energy | 0.00971±0.00442 | 0.03225±0.00865 | 0.03877±0.00598 |  | 0.02354±0.01133 | 0.03028±0.01574 |  | 0.03033±0.01444 | 0.02349±0.01292 |
|  | Probability max | 0.00877±0.00491 | 0.03187±0.00868 | 0.03763±0.00603 |  | 0.02261±0.01137 | 0.02957±0.01581 |  | 0.02948±0.01445 | 0.02271±0.01311 |
|  | Entropy | 0.6±0.03 | 0.44±0.06 | 0.37±0.04 |  | 0.49±0.09 | 0.45±0.12 |  | 0.44±0.11 | 0.49±0.1 |
|  | Correlation | 64450.95±176.47 | 63775.62±538.31 | 62882.03±353.69 |  | 63635.81±683.48 | 63769.92±807.84 |  | 63663.61±801.58 | 63742.13±695.12 |
| GLRLM | LRE | 1.624±0.418 | 4.825±1.423 | 5.694±1.24 |  | 3.417±1.413 | 4.678±2.421 |  | 4.391±2.121 | 3.704±1.98 |
|  | GLN | 0.006±0.004 | 0.031±0.012 | 0.043±0.009 |  | 0.023±0.015 | 0.03±0.02 |  | 0.032±0.019 | 0.021±0.015 |
|  | RLN | 0.055±0.008 | 0.028±0.007 | 0.023±0.004 |  | 0.038±0.014 | 0.033±0.017 |  | 0.032±0.015 | 0.038±0.016 |
|  | LGRE | 0.00002±0.000001 | 0.000017±0.000003 | 0.000016±0.000001 |  | 0.000018±0.000002 | 0.000018±0.000003 |  | 0.000017±0.000003 | 0.000018±0.000002 |
|  | HGRE | 53026.53±2082.66 | 60803.7±1868.69 | 63290.02±987.39 |  | 58745.72±4501.81 | 59334.44±4893.72 |  | 60265.81±4009.08 | 57814.35±5028.59 |

Supplementary material 2. The Computer Features according to the Scanning Parameters in the -630 HU Nodule Phantoms.

|  | | Slice Thickness | | |  | mAs | |  | Reconstruction Algorithm | |
| --- | --- | --- | --- | --- | --- | --- | --- | --- | --- | --- |
|  |  | 5.0 | 2.5 | 1.25 |  | 30 | 120 |  | Lung | Standard |
| Histogram | Mean | -691.88±12.17 | -654.41±15.29 | -649.9±16.86 |  | -664.57±22.49 | -666.23±25.48 |  | -653.72±23.99 | -677.08±17.49 |
|  | Stdev | 58.9±17.14 | 65.01±30.35 | 85.88±51.15 |  | 82.68±44.64 | 57.18±22.19 |  | 93.77±39.16 | 46.09±11.52 |
|  | Variance | 3759.49±2686.01 | 5136.46±4955.39 | 9957.96±11480.89 |  | 8811.42±9867.3 | 3757.85±3549.63 |  | 10313.6±9317.54 | 2255.68±1812.62 |
|  | Skewness | 3.22±0.61 | 4.35±1.12 | 4.61±1.59 |  | 3.67±0.96 | 4.44±1.51 |  | 3.45±0.6 | 4.66±1.55 |
|  | Kurtosis | -0.46±0.26 | -0.76±0.63 | -0.66±0.67 |  | -0.47±0.44 | -0.78±0.63 |  | -0.17±0.21 | -1.08±0.41 |
|  | Energy | 0.03±0.01 | 0.04±0.02 | 0.03±0.02 |  | 0.03±0.01 | 0.04±0.02 |  | 0.02±0.01 | 0.05±0.01 |
|  | Entropy | 5.15±0.38 | 5.18±0.71 | 5.48±0.83 |  | 5.54±0.7 | 5±0.55 |  | 5.81±0.51 | 4.73±0.3 |
| GLCM | Contrast | 22.55±15.91 | 43.21±25.72 | 66.95±44.66 |  | 54.28±43.4 | 34.2±22.54 |  | 63.49±41.12 | 24.99±12.53 |
|  | Dissimilarity | 0.82±0.31 | 1.13±0.49 | 1.45±0.67 |  | 1.31±0.66 | 0.96±0.4 |  | 1.5±0.58 | 0.77±0.22 |
|  | Homogeneity | 0.01414±0.00292 | 0.01344±0.00544 | 0.01135±0.0049 |  | 0.01125±0.00417 | 0.0147±0.00455 |  | 0.00916±0.00273 | 0.01679±0.00271 |
|  | ASM | 0.00002±0.00001 | 0.00002±0.00001 | 0.00001±0.00001 |  | 0.00001±0.00001 | 0.00002±0.00001 |  | 0.00001±0.00001 | 0.00002±0.00001 |
|  | Energy | 0.00429±0.00113 | 0.00419±0.00136 | 0.00369±0.00107 |  | 0.00357±0.0008 | 0.00454±0.00137 |  | 0.00324±0.00071 | 0.00487±0.00107 |
|  | Probability max | 0.00089±0.00054 | 0.00086±0.00056 | 0.00075±0.0003 |  | 0.00066±0.00024 | 0.001±0.00059 |  | 0.00058±0.00023 | 0.00108±0.00054 |
|  | Entropy | 0.61±0.03 | 0.62±0.04 | 0.63±0.04 |  | 0.63±0.03 | 0.61±0.04 |  | 0.64±0.03 | 0.59±0.03 |
|  | Correlation | 64929.14±495.97 | 64486.0±608.16 | 63917.16±1081.56 |  | 64201.68±1026.79 | 64686.52±598.83 |  | 63990.45±985.85 | 64897.75±380.06 |
| GLRLM | LRE | 1.134±0.048 | 1.141±0.101 | 1.111±0.07 |  | 1.1±0.052 | 1.157±0.087 |  | 1.073±0.032 | 1.184±0.067 |
|  | GLN | 0.003±0.001 | 0.003±0.002 | 0.003±0.002 |  | 0.002±0.001 | 0.003±0.002 |  | 0.002±0.001 | 0.004±0.001 |
|  | RLN | 0.066±0.002 | 0.066±0.004 | 0.067±0.003 |  | 0.067±0.002 | 0.065±0.003 |  | 0.068±0.001 | 0.064±0.002 |
|  | LGRE | 0.000062±0.000005 | 0.000056±0.000001 | 0.000057±0.000004 |  | 0.000059±0.000003 | 0.000058±0.000006 |  | 0.000058±0.000004 | 0.000059±0.000005 |
|  | HGRE | 16578.428±655.711 | 18404.459±723.419 | 18745.655±832.318 |  | 18030.869±1150.19 | 17788.159±1251.369 |  | 18488.624±1222.107 | 17330.404±865.241 |

Supplementary material 3. The Computer Features in 12 Different Scan Parameters in 100HU Nodule Phantoms

|  | | Slice Thickness, mAs, Reconstruction Algorithm | | | | | | | | | | | |
| --- | --- | --- | --- | --- | --- | --- | --- | --- | --- | --- | --- | --- | --- |
|  |  | 1.25, 30, Lung | 1.25, 120, Lung | 1.25, 30, Standard | 1.25, 120, Standard | 2.5, 30, Lung | 2.5, 120, Lung | 2.5, 30, Standard | 2.5, 120, Standard | 5.0, 30, Lung | 5.0, 120, Lung | 5.0, 30, Standard | 5.0, 120, Standard |
| Histogram | Mean | 148.92±10.32 | 150.07±5.17 | 42.51±7.47 | 42.83±3.1 | 80.15±19.85 | 90.66±28.18 | -32.51±24.47 | -32±21.47 | -97.54±27.83 | -82.05±13.98 | -161.17±25.89 | -146.75±10.64 |
|  | Stdev | 164.33±19.2 | 121.69±6.41 | 94.17±18.22 | 88.72±3.9 | 188.02±23.62 | 162.25±53.24 | 156.42±19.94 | 152.12±19.05 | 197.68±7.26 | 182.38±7.66 | 196.57±2.98 | 187.76±5.42 |
|  | Variance | 27355.67±6346.87 | 14847.18±1581.28 | 9182.71±3447.93 | 7885.08±691.07 | 35881.86±8858.84 | 29016.53±28247.06 | 24843.83±6222.89 | 23484.41±5800.57 | 39126.19±2876.22 | 33318.97±2794.25 | 38649.92±1174.66 | 35283.21±2048.77 |
|  | Skewness | 4.66±1.13 | 6.51±0.65 | 12.5±1.19 | 13.64±0.75 | 5.81±0.44 | 7.48±1.25 | 5.19±0.69 | 4.64±0.66 | 4.47±1.12 | 4.33±1.18 | 2.76±0.1 | 2.6±0.1 |
|  | Kurtosis | -0.19±0.44 | -0.31±0.24 | -2.71±0.21 | -3.06±0.1 | -1.13±0.3 | -1.43±0.31 | -1.6±0.16 | -1.51±0.17 | -1.14±0.31 | -1.17±0.31 | -0.74±0.05 | -0.72±0.06 |
|  | Energy | 0.56±0.04 | 0.74±0.05 | 0.42±0.04 | 0.54±0.04 | 0.44±0.03 | 0.58±0.05 | 0.21±0.04 | 0.25±0.05 | 0.08±0.04 | 0.09±0.03 | 0.04±0.02 | 0.04±0.02 |
|  | Entropy | 2.12±0.28 | 1.21±0.24 | 2.7±0.25 | 2.22±0.19 | 2.91±0.21 | 2.15±0.21 | 4.35±0.37 | 4.15±0.38 | 5.61±0.41 | 5.38±0.3 | 6.23±0.31 | 6.12±0.18 |
| GLCM | Contrast | 371.62±54.33 | 367.58±59.22 | 364.09±54.84 | 354.23±51.81 | 258.69±19.88 | 221.88±120.01 | 228.54±10.36 | 219.2±13.08 | 144.83±15.99 | 128.94±19.33 | 118.57±20.67 | 110.95±18.15 |
|  | Dissimilarity | 2.41±0.2 | 2.15±0.28 | 2.33±0.24 | 2.25±0.29 | 1.98±0.19 | 1.56±0.65 | 2.09±0.22 | 2.04±0.23 | 1.95±0.04 | 1.79±0.08 | 1.94±0.04 | 1.85±0.08 |
|  | Homogeneity | 0.0369±0.0017 | 0.048±0.0018 | 0.0371±0.0013 | 0.0446±0.0012 | 0.0361±0.0013 | 0.0473±0.0042 | 0.0285±0.0033 | 0.0315±0.0041 | 0.0156±0.0047 | 0.0174±0.0036 | 0.0121±0.0038 | 0.0129±0.0026 |
|  | ASM | 0.0012±0.0001 | 0.0021±0.0002 | 0.001±0.0001 | 0.0018±0.0001 | 0.0011±0.0001 | 0.002±0.0004 | 0.0006±0.0001 | 0.0008±0.0002 | 0.0001±0.0001 | 0.0002±0.0001 | 0.0001±0.0001 | 0.0001±0 |
|  | Energy | 0.0346±0.0021 | 0.0462±0.0026 | 0.0323±0.002 | 0.0419±0.0017 | 0.0327±0.0014 | 0.0448±0.0042 | 0.0235±0.0031 | 0.028±0.0041 | 0.0107±0.0049 | 0.0129±0.0041 | 0.0073±0.0035 | 0.0079±0.0027 |
|  | Probability max | 0.0332±0.0018 | 0.045±0.0023 | 0.031±0.0018 | 0.0413±0.0017 | 0.0322±0.0014 | 0.0444±0.0046 | 0.0231±0.0031 | 0.0278±0.0042 | 0.0099±0.0053 | 0.0121±0.0044 | 0.0062±0.0042 | 0.0069±0.0032 |
|  | Entropy | 0.39±0.02 | 0.32±0.02 | 0.4±0.02 | 0.36±0.01 | 0.42±0.01 | 0.35±0.02 | 0.5±0.02 | 0.48±0.02 | 0.59±0.02 | 0.57±0.03 | 0.62±0.01 | 0.61±0.02 |
|  | Correlation | 62806.3±352.08 | 62904.05±395.32 | 62848.13±351.45 | 62969.65±317.08 | 63615.46±133.45 | 63875.21±1061.98 | 63760.58±78.84 | 63851.23±96.58 | 64315.36±126.57 | 64465.27±168.07 | 64469.04±164.82 | 64554.13±164.1 |
| GLRLM | LRE | 4.52±0.32 | 6.86±0.31 | 4.62±0.35 | 6.77±0.87 | 4.6±0.4 | 6.81±0.64 | 3.55±0.7 | 4.34±1.09 | 1.74±0.47 | 1.81±0.41 | 1.46±0.36 | 1.47±0.31 |
|  | GLN | 0.04±0.004 | 0.055±0.006 | 0.033±0.005 | 0.045±0.006 | 0.036±0.002 | 0.047±0.004 | 0.019±0.003 | 0.023±0.004 | 0.007±0.003 | 0.009±0.003 | 0.003±0.002 | 0.004±0.002 |
|  | RLN | 0.026±0.001 | 0.018±0.001 | 0.027±0.001 | 0.019±0.001 | 0.027±0.001 | 0.019±0.002 | 0.035±0.004 | 0.03±0.005 | 0.054±0.008 | 0.05±0.006 | 0.059±0.007 | 0.058±0.005 |
|  | LGRE | 0.0000164±0.0000005 | 0.0000155±0.0000005 | 0.0000165±0.0000005 | 0.000016±0 | 0.0000169±0.0000004 | 0.0000172±0.0000051 | 0.0000177±0.0000005 | 0.0000175±0.0000005 | 0.0000196±0.0000005 | 0.0000194±0.0000005 | 0.0000214±0.0000005 | 0.0000212±0.0000004 |
|  | HGRE | 62882.48±853.27 | 64308.07±471.5 | 62667.78±937.18 | 63301.75±742.62 | 61906.24±262.13 | 62765.79±1759.99 | 59104.91±712.34 | 59437.85±715.66 | 54805.86±852.14 | 54926.44±784.44 | 51107.07±1186.19 | 51266.76±1007.93 |

Supplementary material 4. The Computer Features in 12 Different Scan Parameters in -630 HU Nodule Phantoms

|  | | Slice Thickness, mAs, Reconstruction Algorithm | | | | | | | | | | | |
| --- | --- | --- | --- | --- | --- | --- | --- | --- | --- | --- | --- | --- | --- |
|  |  | 1.25, 30, Lung | 1.25, 120, Lung | 1.25, 30, Standard | 1.25, 120, Standard | 2.5, 30, Lung | 2.5, 120, Lung | 2.5, 30, Standard | 2.5, 120, Standard | 5.0, 30, Lung | 5.0, 120, Lung | 5.0, 30, Standard | 5.0, 120, Standard |
| Histogram | Mean | -639.03±14.36 | -634.47±14.71 | -661.91±2.69 | -664.21±3.2 | -638.38±4.01 | -640.92±4.52 | -668.33±2.37 | -670.02±2.81 | -683.54±7.19 | -685.99±4.99 | -696.24±3.82 | -701.75±17.11 |
|  | Stdev | 160.92±30.43 | 90.63±21.65 | 51.67±8.09 | 40.28±6.77 | 110.94±18.07 | 65.29±9.59 | 44.45±4.76 | 39.37±5.61 | 78.26±11.28 | 56.59±5.75 | 49.83±4.36 | 50.93±22.11 |
|  | Variance | 26774.81±9847.99 | 8658.84±4823.35 | 2731.77±840.95 | 1666.43±546.73 | 12617.76±4112.58 | 4350.65±1248.95 | 1997.77±424.45 | 1579.65±441.64 | 6245.7±1812.53 | 3233.74±653.43 | 2500.63±434.71 | 3057.8±4136.7 |
|  | Skewness | 2.85±0.21 | 3.63±0.4 | 5.08±0.41 | 6.87±0.45 | 3±0.18 | 3.92±0.66 | 4.7±0.43 | 5.76±0.51 | 3.46±0.4 | 3.84±0.64 | 2.91±0.3 | 2.65±0.12 |
|  | Kurtosis | 0.02±0.1 | -0.17±0.24 | -0.88±0.15 | -1.61±0.2 | -0.02±0.11 | -0.38±0.19 | -1.05±0.13 | -1.6±0.14 | -0.23±0.17 | -0.24±0.16 | -0.66±0.09 | -0.7±0.1 |
|  | Energy | 0.01±0 | 0.02±0 | 0.04±0.01 | 0.06±0.01 | 0.02±0 | 0.03±0 | 0.05±0.01 | 0.07±0.01 | 0.02±0 | 0.03±0 | 0.04±0 | 0.04±0.01 |
|  | Entropy | 6.59±0.21 | 5.82±0.23 | 5.03±0.22 | 4.47±0.19 | 6.17±0.24 | 5.41±0.22 | 4.79±0.18 | 4.37±0.16 | 5.67±0.21 | 5.18±0.15 | 4.97±0.15 | 4.76±0.16 |
| GLCM | Contrast | 133.01±27.57 | 69.1±21.59 | 35.79±1.58 | 29.9±0.68 | 80.87±19.72 | 43.02±8.74 | 25.97±5.31 | 22.99±4.63 | 34.39±11.12 | 20.55±7.91 | 15.64±5.92 | 19.63±24.89 |
|  | Dissimilarity | 2.43±0.28 | 1.59±0.28 | 1±0.07 | 0.79±0.03 | 1.85±0.26 | 1.19±0.17 | 0.8±0.12 | 0.67±0.11 | 1.14±0.19 | 0.8±0.13 | 0.65±0.11 | 0.7±0.41 |
|  | Homogeneity | 0.005767±0.000411 | 0.008342±0.001061 | 0.013053±0.001328 | 0.018248±0.001189 | 0.006842±0.000921 | 0.010459±0.001237 | 0.015674±0.001356 | 0.020765±0.00136 | 0.010066±0.000938 | 0.013493±0.001046 | 0.016122±0.000993 | 0.016867±0.001639 |
|  | ASM | 0.000008±0.000001 | 0.000009±0.000003 | 0.000015±0.000003 | 0.000028±0.000006 | 0.000008±0.000002 | 0.000011±0.000003 | 0.00002±0.000005 | 0.000038±0.000008 | 0.000012±0.000004 | 0.000018±0.000008 | 0.000019±0.000005 | 0.00003±0.000016 |
|  | Energy | 0.0027±0.00024 | 0.0029±0.00040 | 0.0037±0.00044 | 0.0052±0.00057 | 0.0028±0.00039 | 0.0033±0.00046 | 0.0043±0.00054 | 0.0061±0.00067 | 0.0033±0.00062 | 0.0041±0.00089 | 0.0042±0.00062 | 0.0053±0.00123 |
|  | Probability max | 0.000701±0.000289 | 0.000497±0.000136 | 0.000694±0.000129 | 0.001091±0.000241 | 0.00042±0.00008 | 0.000526±0.00011 | 0.000821±0.000214 | 0.001678±0.000472 | 0.000527±0.000153 | 0.000825±0.000288 | 0.000807±0.000248 | 0.001408±0.000776 |
|  | Entropy | 0.66±0.02 | 0.65±0.02 | 0.62±0.02 | 0.58±0.02 | 0.66±0.02 | 0.64±0.02 | 0.6±0.02 | 0.57±0.02 | 0.64±0.03 | 0.61±0.03 | 0.61±0.02 | 0.58±0.03 |
|  | Correlation | 62352.99±704.06 | 63872.42±643.92 | 64645.73±33.85 | 64797.5±16.47 | 63588.09±466.22 | 64505.91±199.76 | 64883.64±113.85 | 64966.41±95.63 | 64622.93±304.16 | 65000.39±212.72 | 65116.74±156.28 | 64976.52±846.85 |
| GLRLM | LRE | 1.057±0.022 | 1.06±0.019 | 1.113±0.029 | 1.213±0.04 | 1.041±0.016 | 1.08±0.022 | 1.154±0.032 | 1.287±0.062 | 1.079±0.016 | 1.119±0.025 | 1.157±0.033 | 1.182±0.033 |
|  | GLN | 0.001±0 | 0.002±0 | 0.003±0 | 0.005±0.001 | 0.001±0 | 0.002±0 | 0.003±0 | 0.005±0 | 0.002±0 | 0.003±0 | 0.003±0 | 0.004±0.002 |
|  | RLN | 0.069±0.001 | 0.069±0.001 | 0.067±0.001 | 0.063±0.001 | 0.07±0.001 | 0.068±0.001 | 0.065±0.001 | 0.061±0.002 | 0.068±0.001 | 0.066±0.001 | 0.065±0.001 | 0.064±0.001 |
|  | LGRE | 0.000061±0.000006 | 0.000055±0.000005 | 0.000056±0.000001 | 0.000056±0 | 0.000056±0.000001 | 0.000054±0.000001 | 0.000057±0 | 0.000057±0 | 0.000061±0.000001 | 0.000061±0.000002 | 0.000062±0.000001 | 0.000064±0.000011 |
|  | HGRE | 19660.528±326.913 | 19340.641±539.905 | 18045.883±96.739 | 17935.568±112.905 | 19240.944±222.775 | 18960.657±126.284 | 17722.166±132.667 | 17694.069±96.857 | 17026.012±231.068 | 16702.965±434.335 | 16489.679±219.721 | 16095.057±1007.949 |
